# Is There a Role for Molecular Testing for Low-Risk Differentiated Thyroid Cancer? A Cost-Effectiveness Analysis

**DOI:** 10.3390/cancers15030786

**Published:** 2023-01-27

**Authors:** Idit Tessler, Moshe Leshno, Gilad Feinmesser, Eran E. Alon, Galit Avior

**Affiliations:** 1 Department of Otolaryngology Head and Neck Surgery, Sheba Medical Center, Ramat Gan 52621, Israel; 2 Faculty of Medicine, Tel Aviv University, Tel Aviv 6423906, Israel; 3 Coller School of Management, Tel Aviv University, Tel Aviv 6423906, Israel; 4 Faculty of Medicine, Technion, Haifa 3200003, Israel

**Keywords:** thyroid cancer, molecular testing, molecular diagnostic, thyroid surgery

## Abstract

**Simple Summary:**

Molecular testing for thyroid nodules aims to predict malignancy. However, its role in guiding treatment decisions is not yet fully understood. The long-term nature of thyroid cancer and the relatively high cost of genetic tests have made it challenging to study the use of molecular testing for cancer management in real-life situations. To address this, we developed a computer model that simulated the use of molecular testing to guide treatment in patients with low-risk differentiated thyroid cancer (lrDTC) and compared it to a standard treatment approach. We found that the strategy of including molecular testing in the pre-operative evaluation was cost effective, leading to a gain of 1.7 quality-adjusted life years at an additional cost of $327 per patient compared to the standard approach. This suggests that molecular testing for lrDTC may be a worthwhile investment that can improve patient outcomes by allowing for personalized treatment based on an individual’s personal risk level.

**Abstract:**

Molecular testing for thyroid nodules has been rapidly developed in recent years, aiming to predict the presence of malignancy and aggressive features. While commonly utilized to predict malignancy, its role in guiding the management approach is still developing. The high cost of genetic tests and long-term sequences of thyroid cancer is limiting to real-life studies. **Objective**: To evaluate the cost effectiveness of molecular testing for low-risk differentiated thyroid cancer (lrDTC). **Methods**: We developed a Markovian decision tree model of a simulated lrDTC cohort, comparing two management strategies: *(I) Conducting genetic tests (GT)*—patients are stratified into three risk groups for distant metastasis by the identified molecular markers: low-, intermediate- and high-risk molecular profile; followed by management accordingly: patients with low-risk will undergo hemithyroidectomy (HT), patients with intermediate-risk will undergo total thyroidectomy (TT), and high-risk patients will undergo TT with central neck dissection; *(II) Without genetic tests (wGT)*—all patients will undergo HT according to the ATA recommendations for lrDTC. Outcomes were measured as quality-adjusted life years (QALYs) and costs of each strategy. **Results:** GT was found as cost effective, leading to a gain of 1.7 QALYs with an additional cost of $327 per patient compared to wGT strategy. This yielded an incremental cost-effectiveness ratio of $190 per QALY. Sensitivity analysis demonstrated robust results across the variables’ ranges. The most impactful variable was the benefit from performing TT rather than HT for intermediate to high-risk patients. **Conclusions:** Our model found that molecular testing for lrDTC is cost-effective, allowing tailored management according to the patient’s personal risk level reflected in the genetic profile, hence improving outcomes.

## 1. Introduction 

Thyroid cancer is the most common endocrine malignancy with continuously increasing incidence [1]. The vast majority of thyroid cancers are epithelial follicular cell-derived thyroid cancers, classified as differentiated thyroid cancer (DTC) [2,3]. In most patients, DTC is an indolent and curable disease. However, in some cases, the disease could have an aggressive course with high recurrence rates (10–30%), distance metastasis and resistance to conventional treatment [4]. Early identification of these cases is crucial to tailor timely preventative management. DTC is usually first diagnosed by fine-needle cytology [5,6]. Once diagnosed, the decision regarding the extent of surgery (hemithyroidectomy or total thyroidectomy) is required. Traditional clinical risk factors are used to facilitate the decision, including the patient’s age and sex, preoperative imaging variables such as tumor size, extrathyroidal extension ETE, and lymph node and/or distant metastases. Detection of these variables preoperatively is challenging as it is highly dependent on the patients’ lesions, and operator factors, which might lead to inaccurate preoperative assessment [7].

Molecular profiling has rapidly evolved in the past decade, showing promising results in thyroid cancer risk stratification [8]. While initially used to rule-out malignancy, several genetic variant mutations are now considered markers of level of tumor behavior aggressiveness. Mutations in PIK3CA, TERT, P53, ALK, and BRAF genes suggests a more aggressive disease. In contrast, other variants are commonly associated with more indolent courses, such as RAS mutations [9,10]. Molecular profiling is now commonly used worldwide and was incorporated into the guidelines. It is primarily used for malignancy detection in indeterminate nodules (Bethesda III to V), but with rising use for deciding on surgery extension for DTC [11]. Prior cost-effectiveness studies have shown the beneficial economic effect of thyroid genetics testing, yet studies referred to its use for indeterminate nodules (Bethesda III and IV) [12,13,14].

Cost-effectiveness analysis is a tool used in medicine to compare the relative costs and benefits of different treatment options. It is often used in clinical decision making to determine the most cost-effective course of action, given a specific set of circumstances. This type of analysis is typically performed by creating a mathematical model that simulates the use of different treatments and estimates the costs and outcomes associated with each option. The results of a cost-effectiveness analysis can be expressed in terms of a cost-effectiveness ratio, which compares the difference in cost between two options to the difference in outcomes they produce.

In this article, we perform a cost-effectiveness analysis to evaluate the use of molecular testing in the treatment of low-risk differentiated thyroid cancer. Specifically, we will compare the costs and benefits of using molecular testing to guide treatment decisions to a standard treatment approach. Our goal is to determine if molecular testing is a cost-effective option for low-risk DTC (lrDTC).

## 2. Methods

### 2.1. Model Structure

A decision tree analytic model with a state transition Markov model was constructed to estimate differences in health benefits and costs as a consequence of the decision to perform, or not to perform, molecular testing for lrDTC.

Our analysis methodology and results are reported according to the Consolidated Health Economic Evaluation Reporting Standards (CHEERS) guidelines, that were created for reporting evaluation of economic health interventions of.. These guidelines provide a set of recommendations for presenting the results of cost-effectiveness analyses in a comprehensive and transparent manner, allowing readers to fully understand and evaluate the findings.

We used TreeAge PRO 2017 software (TreeAge Software, Williamstown, MA, USA) to create the model. The TreeAge software is a specialized computer program designed for conducting cost-effectiveness analyses. It allows users to build mathematical models that simulate the use of different treatments and estimate the costs and outcomes associated with each option. In this study, the TreeAge PRO software was used to model the incremental cost-effectiveness ratio (ICER), which is a measure of the cost effectiveness of one treatment option compared to another. The ICER is calculated by dividing the difference in cost between two options by the difference in outcomes they produce.

Additional analyses were performed using MATLAB (MathWorks, 2020b).

The model is based on a simulated cohort. For each strategy, we calculated the cost and clinical probabilities of possible outcomes and the resulting health benefits. Clinical probabilities were derived from previous literature.

Health benefits were used as the main outcome, and were measured using quality-adjusted life years (QALYs). QALY is a measure of both life quality and quantity. It is calculated using utility weights, which scale the life quality in each health state ranging from 1 (best attainable health) to 0 (death). The utility weights are then multiplied by life years to result in the QALY.

### 2.2. Clinical Data and Probabilities

**Figure 1:**
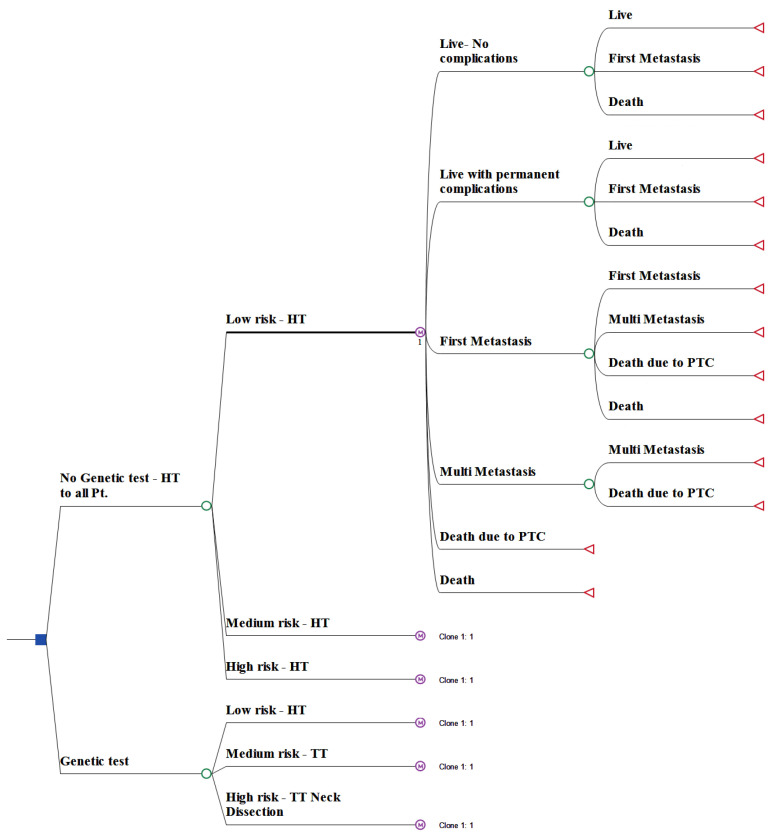
The model decision tree. The model comparing the results of two optional management strategies for low-risk differentiated thyroid cancer: without vs. with the performance of genetic testing. The variable definitions are detailed in Table 1. PTC: papillary thyroid carcinoma; HT: hemithyroidectmy; TT: total thyroidectomy; Clon 1: represents the same decision tree branches as shown following the number 1.

The course of each strategy is described in the decision model (Figure 1). A decision tree is a graphical representation of a series of decisions and their possible consequences, which is used to model the potential outcomes of each strategy. A Markovian model was incorporated to describe the probability of transitioning from one state to another in the decision tree over time. The role of Markov models is to model processes that involve a sequence of events, where the likelihood of future events is dependent only on the current state and not on the past states.

Our model is based on a simulated cohort of 80% female patients diagnosed with lrDTC, with a base case of 45 years old. All patients have a low-risk DTC, which is defined as a size less than 4 cm, with no evidence of extra-capsular extension or lymph-node metastasis.

We compared two optional management strategies:

(i) **Conducting genetic testing (GT)**—patients are stratified into three molecular risk groups [25]: (1) the low-risk molecular group: included RAS and RAS-like alterations without additional mutation; (2) the intermediate-risk group included BRAF V600E, which is the most common variant, other BRAF-like alterations, and copy number alteration (CNA); (3) The high-risk group including TERT, TP53, AKT1, and PIK3CA and other early mutations and a late-hit mutation [26].

Patients are managed according to the genetic risk: patients with low risk will undergo hemi-thyroidectomy (HT), patients with intermediate risk will undergo total thyroidectomy (TT) and patients with high risk will undergo TT with neck dissection (ND).

(II) **Without genetic testing (wGT)**—the second strategy is without performing molecular testing; hence, all patients will undergo hemithyroidectomy according to the ATA recommendations for lrDTC.

### 2.3. Clinical Course

Our model used the predictions of the risk of developing distant metastasis (DM), stratified by each genetic profile. Within the 5% prevalence of DM expected in most populations of patients with DTC [4], the probability of DM would be from 0.2% in the low-risk molecular profile, 4.7% in the intermediate-risk molecular profile, and 19.3% in the high-risk molecular profile [25].

### 2.4. Costs

The model was constructed from the perspective of the United States (Medicare) healthcare system. The average thyroid surgery cost by Medicare which includes all the required resources (such as operating room time, anesthesia, and professional fees) was as follows: $3538 for lobectomy, $4102 for total thyroidectomy, $7731 for neck dissection. The average Medicare cost for ThyroSeq^®^ was $4056 per sample. Costs were discounted at a rate of 3% per year. A willingness to pay threshold of $50,000 per QALY was used. Costs and range for sensitivity analyses are presented in Table 1.

### 2.5. Sensitivity Analysis

Sensitivity analyses were performed in order to examine the robustness of the results considering possible uncertainties and real-life variation. This allows us to include a range in the model, rather than a single value, for each variable. One-way sensitivity analysis (involving varying the value of a single input variable in the model to see how it affects the results) was conducted on each of the model inputs. The possible values range of each probability for clinical event was derived from previous literature, and are presented in Table 1. A range of ± 20% was calculated for all costs, and a range of ±30% was considered for utility values. A tornado diagram was plotted to show the relative impact of different variables on the cost-effectiveness, and how the range of these variables affect the overall cost-effectiveness of the model.

To aggregate uncertainty from multiple variables at the same time, we also performed a probabilistic sensitivity analysis. Probabilistic sensitivity analysis involves simulating a large number of random variations in the input variables and analyzing the resulting distribution of results.

We used different types of distributions for the probabilistic sensitivity analysis: Gamma for costs, beta or triangular for model probabilities, and beta for health utilities. A probabilistic sensitivity analysis was run for 100 trials of 10,000 samples. In addition, a cost-effectiveness acceptability curve was constructed to represent the probability of the model strategies’ cost-effectiveness across a range of willingness-to-pay thresholds.

## 3. Results

In the base case analysis, GT was found as cost effective, leading to a gain of 1.7 QALYs with an additional cost of $327 per patient compared to the wGT strategy. This gain yielded an incremental cost-effectiveness ratio (ICER) of $190 per QALY (Table 2), i.e., GT can improve QALY, and in order to gain an additional 1 year of maximal quality of life by using GT, the cost will be $190. This cost is substantially lower than the accepted threshold that is considered as economically beneficial to pay per QALY ($50,000).

### Sensitivity Analysis

**Figure 2:**
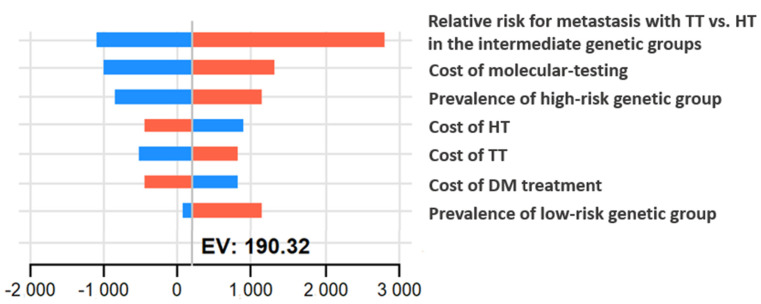
Tornado diagram presenting one-way sensitivity analysis of incremental cost effectiveness of GT over wGT. The vertical line represents the ICER using base-case values. Color coding represents the range of each variable from low values (blue) to high values (red). This demonstrates the effect of possible variation in the variable value on the ICER: increasing values are indicated by red bars, while the effect of decreasing is indicated by blue bars. Prices are in USD. TT: total thyroidectomy; HT: hemithyroidectomy; DM: distant metastasis; ICER: incremental cost effectiveness ratio; EV: expected value.

A one-way sensitivity analysis conducted to assess the impact of the models’ variables on the ICER demonstrated that GT remained cost effective for all cost, utility, and probability variables examined. The variables with the greatest influence on cost, effectiveness, and ICER are presented in the tornado diagram (Figure 2).

The benefit from performing TT vs. HT on the risk for DM in the intermediate-risk molecular group was the found to have the greatest impact on resulting ICER. Even with the lowest value we considered for the relative risk, the results still benefit from the performance of genetic testing, which supports the model’s robustness. The prevalence of high and low-risk genetic profiles among the genetic risk group was also found to affect the model results. While for the high-risk group a more extensive surgery may improve outcome, the management of the low-risk population does not change following molecular testing; hence, it adds to the model the genetic costs without an effectiveness benefit.

**Figure 3:**
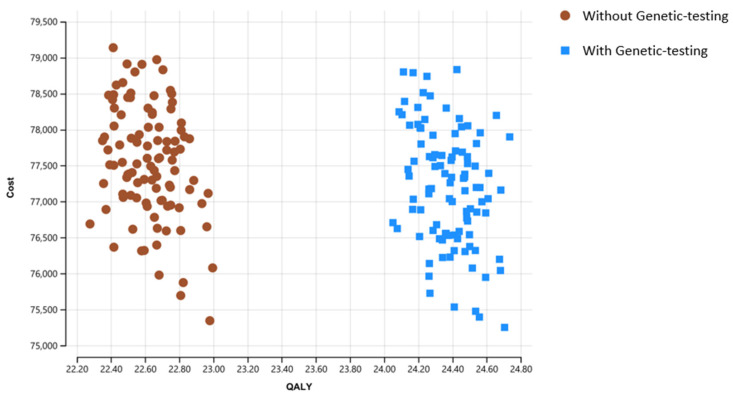
Cost-effectiveness probabilistic sensitivity analysis of GT over wGT demonstrating cost and effectiveness of the models’ strategies over 10,000 iterations with simultaneous variation of input values. In the scatterplot each dot represents a single simulated cohort within the possible range of the variable’s values. The X axis represented QALYs gained, and Y axis represent the overall costs. In all iterations GT resulted higher QALY, mostly in higher cost. GT: genetic testing; QALY: quality-adjusted life years.

**Figure 4:**
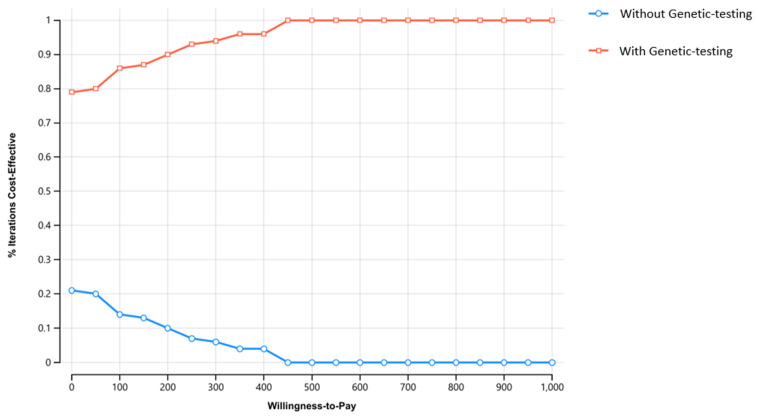
Cost effectiveness acceptability curve. The Y axis represent willingness-to-pay threshold ($ to QALY). The Y axis represents the precect of iteration. QALY: quality-adjusted life-year; WTP: willingness to pay.

The cost of the genetic test had a substantial influence both on incremental cost and ICER (Figure 2); however, through the full range of tests costs, GT remains the preferred strategy over wGT. Of note, we consider a very wide range for this cost, reflecting the dynamic seen in recent years in genetic technologies. Results of the probabilistic sensitivity analysis are presented in Figure 3 and Figure 4. On probabilistic sensitivity analysis, through all iterations GT resulted in higher QALYs.

## 4. Discussion

To the best of our knowledge, the current study is the first to evaluate the cost effectiveness of utilizing a molecular testing strategy for the management decision-making for lrDTC, compared to the current guidelines’ recommendation. In this simulated model, GT strategy was robustly found to result in QALYs gained with ICER of $190.

DTCs are mostly indolent cancers with limited morbidity or mortality [27]. Identifying the minority of the cases with aggressive behavior is critical yet challenging. Clinical and histological grading are currently used; however, 5-year relative survival decreases from 99.9% to 55% when DTC occurs with DM [4]. An accurate preoperative diagnosis could potentially identify at-risk patients for aggressive diseases and allow initial appropriate surgical management.

Molecular testing has rapidly evolved in recent years [8,28,29]. In addition to its established performance for cancer risk stratifications, studies have now shown an association between the behavior of thyroid cancer and the level of aggressiveness to the molecular profile [25,30]. In a retrospective case-control study, Yip et al. demonstrated that molecular-based classification could accurately stratify the risk of DM [25]. This has the potential to target high-risk patients and tailor treatment.

However, the performance of prospective studies for the clinical and economic effects of such practice is challenging. Due to the indolent nature of thyroid cancer, long-term follow-up is crucial to assess the effectiveness of molecular testing and the chosen management approach. In addition, molecular tests still involve high costs, which may limit use [31].

Previous cost-effectiveness studies which have evaluated the use of molecular testing have focused on Bethesda III/IV nodules, comparing the use of molecular testing versus diagnostic thyroid lobectomy. Initially, studies showed debatable results; however, recent studies support the cost effectiveness of this practice [12,13,14,32,33].

Our model presents the sequencing resulting from the risk of developing DM. The model is based on the assumption that aggressive surgical intervention will reduce the risk of developing DM. The role of prophylactic central compartment ND is controversial. Supporting evidence suggests improved disease-specific survival and local recurrence [19,34]. The 2015 ATA guidelines state that the value of ND for an individual patient depends upon the utility of the staging information [20]. Genetic data may provide the required stratification to target high-risk populations, hence increasing the benefit of prophylactic ND.

Genetic testing for thyroid cancer is a relatively new and developing field. We found that the tests cost was a main factor in the model results. The cost of these tests has been a significant barrier to their widespread adoption. However, recent advancements in technology have led to a decrease in the cost of these panels. As the cost of these tests continues to decrease, they may become a more viable option for routine use in clinical practice worldwide.

Molecular testing for thyroid nodules not only provides patients with a more accurate diagnosis and personalized treatment plan, but also has a significant emotional impact. While the diagnosis of thyroid cancer can be emotionally distressing, molecular testing can provide patients with a sense of reassurance by giving them a better understanding of their condition and the risks they face.

While we considered DM as the primary outcome, locoregional recurrence can also impact on the costs in cases of aggressive disease which was initial treated with HT. By adopting a conservative approach and thus avoiding complexity, we did not include these possible costs, which would presumably increase genetic screening effectiveness.

Radioactive iodine administration is another management option that could be applied with early identification of high-risk cases and improve prognosis [35]. To avoid an unwieldy model, this option was not incorporated into our model. Nevertheless, it has the potential to increase the benefit of genetic testing.An additional limitation of this study is that we did not account for possible variations due to the use of different genetic panels. Finally, the design of the cost-effectiveness analysis is a theoretical study based on costs, probabilities, and assumptions derived from evidence-based data and may not represent all variations. Yet, our findings can reassure and support clinicians in further real-life studies.

## 5. Conclusions

In conclusion, our model suggests that preoperative molecular testing for lrDTC is cost effective, allowing to tailor management according to the patient’s risk level. Due to the long-term surveillance required to evaluate outcomes in thyroid cancer, this model provides essential evidence to the utility of genetic testing in thyroid cancer and its clinical and economic benefits.

## Figures and Tables

**Table 1:** The model’s probabilities and costs variables.

Variable	Base	Low	High	Refs
Age	45	40	50	
Cost of treatment in distance metastases	22,008	17,500	26,500	[15]
Cost of thyroid lobectomy surgery	3538	2000	5000	[16]
Cost of neck dissection surgery	7731	1000	4000	[16]
Cost of genetic testing panel	4056	2000	6000	[16]
Cost of total thyroidectomy surgery	4102	2500	5500	[16]
Rate of female gender	0.8	0.7	0.9	
Rate of first metastasis in high-risk genetic group	0.193	0.1	0.3	
Rate of first metastasis in intermediate-risk genetic group	0.047	0.01	0.1	
Rate of first metastasis in low-risk genetic group	0.002	0.001	0.01	
Prevalence of high-risk genetic group	0.15	0.01	0.3	[17]
prevalence of low-risk genetic group	0.23	0.2	0.4	
Rate of death in first distant metastasis	0.05	0.02	0.1	[18]
Rate of transition from first metastasis to multi-distant metastasis	0.566	0.4	0.7	[18]
Rate of death in multi-distant metastasis	0.35	0.3	0.4	[18]
Relative risk for the rate for first metastasis of TT with ND vs. HT in high-risk genetic group	0.6	0.5	0.95	[19,20,21,22,23]
Relative risk for the rate for first metastasis of TT vs. HT in intermediate-risk genetic group	0.8	0.5	0.95	[19,20,21,22,23]
Time horizon (Years)	100	90	110	Assumption
Utility of Metastasis	0.8	0.5	0.9	[24]

**Table 2:** Models’ results—cost and effectiveness of genetic testing for lrDTC.

Strategy	Cost	Incremental Cost	Effectiveness	Incremental Effectiveness	ICER
Without genetic testing	75,103		22.256		
With genetic testing	75,430	327	23.972	1.716	190

Costs in USD; ICER: incremental cost-effective ratio.

## Data Availability

All datasets generated during the current study are available from the corresponding author on reasonable request.
